# Diffuse fibrosis and repolarization disorders explain ventricular arrhythmias in Brugada syndrome: a computational study

**DOI:** 10.1038/s41598-022-12239-9

**Published:** 2022-05-20

**Authors:** Niccoló Biasi, Paolo Seghetti, Alessandro Tognetti

**Affiliations:** 1 Department of Information Engineering, University of Pisa, Pisa, Italy; 2 Institute of Life Sciences, Scuola Superiore Sant’Anna, Pisa, Italy; 3 National Research Council, Institute of Clinical Physiology, Pisa, Italy; 4 Research Centre “E. Piaggio”, University of Pisa, Pisa, Italy

**Keywords:** Biomedical engineering, Computational models, Arrhythmias

## Abstract

In this work, we reported a computational study to quantitatively determine the individual contributions of three candidate arrhythmic factors associated with Brugada Syndrome. In particular, we focused our analysis on the role of structural abnormalities, dispersion of repolarization, and size of the diseased region. We developed a human phenomenological model capable of replicating the action potential characteristics both in Brugada Syndrome and in healthy conditions. Inspired by physiological observations, we employed the phenomenological model in a 2D geometry resembling the pathological RVOT coupled with healthy epicardial tissue. We assessed the insurgence of sustained reentry as a function of electrophysiological and structural abnormalities. Our computational study indicates that both structural and repolarization abnormalities are essential to induce sustained reentry. Furthermore, our results suggest that neither dispersion of repolarization nor structural abnormalities are sufficient on their own to induce sustained reentry. It should be noted how our study seems to explain an arrhythmic mechanism that unifies the classic repolarization and depolarization hypotheses of the pathophysiology of the Brugada Syndrome. Finally, we believe that this work may offer a new perspective on the computational and clinical investigation of Brugada Syndrome and its arrhythmic behaviour.

## Introduction

The Brugada syndrome (BrS) is an inherited cardiac disorder, first described by Pedro and Josep Brugada as a specific disease with a characteristic electrocardiographic pattern^1^. The syndrome is associated with ventricular cardiac arrhythmias and sudden cardiac deaths. Indeed, BrS is believed to be responsible for at least 4% of all sudden deaths and at least 20% of sudden deaths in patients without apparent morphological abnormalities^2^. Furthermore, a recent epidemiological study suggested the specific worldwide prevalence of Brs is about 0.05%^3^. The BrS is associated with a vast genetic background, with the most frequent mutations observed in the sodium channel as loss of function mutations (e.g., SCN5A)^4^. Notably, other mutations include loss of function mutations of calcium channel and gain of function mutations of the transient outward potassium current^5^. Currently, there are 18 genes for which mutation is implied in the genesis of BrS, with SCN5A being the most frequent^6,7^. The arrhythmogenesis in BrS is supposed to originate from the Right Ventricular Outflow Tract (RVOT), a thin walled tubular structure positioned between the pulmonary trunk and the right ventricular cavity^8^. Moreover, electrophysiological studies reveal that the RVOT is at the center of the disease process which underlies Brugada syndrome^9^. Clinical and experimental studies revealed that BrS only affects the epicardial layer of the RVOT^8–10^. Indeed, epicardial electrograms recorded in BrS patients show alterations only in the RVOT, whereas endocardial electrograms did not show any pathological sign^11^. BrS electrocardiographic manifestations can be divided into two patterns. Type I pattern consists of J-point-elevation and an inverted T-wave, whereas Type II pattern is characterized by saddleback-type ST-segment elevation followed by either a positive or biphasic T-wave^12^. There are two classical interpretations of the ECG features and pathophysiological mechanism of the BrS: the repolarization disorder theory and the depolarization disorder theory. The repolarization disorder theory attributes the ECG changes to nonuniform alteration of the epicardial right ventricular action potential duration (APD). In particular, the reduced sodium current combined with increased transient outward current may lead to loss of action potential (AP) dome in the epicardium. The loss of action potential dome in the RVOT epicardium is thought to determine the reentrant mechanism in the form of phase-2 reentry (P2R). The repolarization disorder theory is supported by experimental studies on canine right ventricular wedge preparations treated with specific channel blockers to mimic BrS^13–15^. Concerning the depolarization disorder theory, the reduction of sodium current may lead to a significant conduction slowing in the RVOT epicardium, thereby resulting in ST segment elevation^16,17^. Moreover, cardiac structural abnormalities (e.g., fibrosis) are observed in the RVOT of Brugada patients^10,16^ and predispose to excitation failure (i.e., current-load-mismatch), which represents another possible cause of ST-segment elevation^18,19^. Discontinuous conduction through a fibrotic myocardium is reflected in late potentials and fragmented epicardial electrograms, typically observed in BrS patients, supporting the depolarization disorder and current-load-mismatch theories^6,10^. Disruption and slowing of depolarization propagation due to structural abnormalities are thought to be a key element in the genesis of arrhythmic events in BrS^20,21^.

The picture that emerges from the literature, see for reference the works of Hoogendijk et al.^22,23^, is that arrhythmogenesis in BrS may be due to a combination of electrophysiological and structural factors that affect the RVOT (dispersion of repolarization, slowing of conduction, fibrosis). To quantitatively determine each individual contribution to arrhythmogenesis, in this work we performed a computational study on a two-dimensional model of the RVOT enclosed in healthy epicardial tissue. In particular, we studied the insurgence of sustained reentry as a function of electrophysiological and structural alterations of the ROVT. To describe the electrophysiological alterations we adapted our previously published phenomenological model of ventricular epicardial cells^24^ to reproduce the characteristics commonly associated to BrS action potentials, such as loss of AP dome, delayed dome, and low upstroke velocity, which is related to conduction slowing in Brugada patients^16,17^. The simple formulation of the model (i.e. only 3 state variables) allows for fast and efficient simulations while maintaining an accurate representation of the human epicardial AP. Moreover, the low number of parameters (i.e, 17 parameters) facilitates the process of fitting the model to specific pathological alterations. To preliminary determine the different modalities of P2R between healthy and pathological tissue, we developed a 1D cable model of BrS. Notably, our model was able to reproduce antidromic and orthodromic P2R, and delayed or lost dome AP depending on the membrane state. Then, we developed the 2D tissue model of BrS including both altered electrophysiological properties and fibrosis in the region resembling the RVOT. We evaluated the insurgence of sustained reentry for different percentages of fibrotic tissue, strengths of transient outward current, and sizes of the pathological region. The results of our computational study suggest that the presence of fibrotic tissue is an essential component of the arrhythmogenic behaviour of BrS. Nevertheless, we observed that loss of AP dome is also necessary for the genesis of arrhythmic events. Furthermore, the size of the pathological tissue has shown to be meaningful in determining the arrhythmic substrate of BrS. Previous computational studies focused on the genesis of arrhythmic events in BrS^25–29^. However, none of these studies considered structural abnormalities in the pathological tissue that, according to our results, is an essential factor for the onset of arrhythmic events.

## Methods

### Ventricular myocyte model

We developed a phenomenological model capable of replicating the characteristics of healthy and BrS myocytes. We conceived the model to reproduce the characteristics of BrS action potentials, such as lost or delayed dome and low upstroke velocity. To develop the myocyte model we extended the phenomenological model of epicardial tissue described in our previous work^24^. The model represents the total transmembrane current divided into three contributions corresponding to the excitatory, recovery, and transient outward currents. To more accurately represent the AP dome associated with BrS, we modified the original formulation^24^ by introducing a quadratic dependency between the parameter *A* and the variable *u*:

1 $$\begin{aligned} A=A_0+A_1 u^2 \end{aligned}$$

where we added the parameter $A_1$. Note that, by setting $A_1=0$, this formulation is equivalent to the original model (i.e., $A_1=0$ was used for the healthy myocyte in this work). Furthermore, we modified the parameters of the original formulation^24^ to simulate the AP associated with BrS. First, we reduced the values of $\gamma _0$ and $\gamma _1$ to reproduce the slower upstroke dynamics. Indeed, recent studies demonstrated that human induced pluripotent stem cells-derived cardiomyocytes carrying loss-of-function mutation of $Na_v 1.5$ show reduced upstroke velocities^30,31^. Similar results were also obtained in canine RV wedge preparations, where BrS was pharmacologically induced^13^. Moreover, reduced upstroke velocity contributes to conduction slowing, which has been observed in Brugada patients^16,17^. Second, the intensity of the transient outward current was modulated through the parameter $d_w^0$. In particular, a lower value of $d_w^0$ implies a stronger transient outward current. Parameter values for the BrS model were selected to reproduce the AP morphology shown by *in vivo* human BrS epicardial recording of monophasic action potentials^32,33^. Parameter values for healthy and BrS models are reported in Table 1.

**Table 1:** Model parameters.

Parameter	Healthy epicardial	BrS
k	1 m s$^{-1}$	1 m s$^{-1}$
$c_1$	2.6	2.6
$c_2$	1	1
$c_3$	0.5	0.5
a	0.18	0.18
$A_0$	135 mV	90 mV
$A_1$	0 mV	500 mV
B	− 85 mV	− 85 mV
$e_1^0$	0.0059	0.0059
$e_2$	0.015	0.015
$\gamma _0$	8	3
$\gamma _1$	20	7.5
$\alpha$	15	15
$\theta _u$	0.2	0.2
$g_0$	0.1	0.1
$u_M$	0.58	0.58
$e_w^0$	0.04	0.06
$d_w^0$	0.6	0.3–0.6

### Numerical methods

To simulate AP propagation, we incorporated the ventricular model described in the previous section in the monodomain formulation of cardiac tissue:

2 $$\begin{aligned} \frac{\partial V_M}{\partial t}-\nabla \cdot \left( D \nabla V_M\right) =-I_{ion}+I_{ext} \end{aligned}$$

where $I_{ext}$ indicates an external stimulation current. The diffusion coefficient *D* was set to 1.171 cm$^2$ s. This value is derived by Orovio et al.^34^ from experimental measurements of surface-to-volume ratio, cytoplasm resistivity, membrane capacitance, and surface area of human ventricular cells. Temporal integration was performed using an explicit Euler scheme with a time step of $\Delta t=0.02$ ms. Spatial derivatives were approximated with standard second-order finite differences with a spatial resolution of $\Delta x=0.02$ cm. In all the numerical simulations no-flux boundary conditions were adopted. Moreover, each simulation was initialized with the whole tissue in the resting state (i.e., $V_M=-85$ mV, $u=0$, $w=0$). Cardiac tissue was stimulated with strength twice the diastolic threshold for 2 ms.

To assess the BrS myocyte model, we evaluated the AP morphology in a 2 cm long homogeneous cable model of BrS myocytes for different values of $d_w^0$ (i.e., different intensity of the transient outward current). Then, we studied the mechanism of P2R in a one-dimensional model of BrS represented by a 10 cm long cable model composed of two regions: one with the BrS myocytes (i.e., the BrS region) and the other with the healthy tissue. To assess how P2R is influenced by the intensity of the transient outward current, we performed simulations with different values of $d_w^0$ for the BrS region.

We performed a two-dimensional simulation on a $10 \times 10$ cm square in which a Brugada zone resembling the RVOT epicardium is enclosed in the healthy epicardial tissue. We represented the BrS zone as a semicircle showing both electrophysiological alterations and diffuse fibrosis. We introduced diffuse fibrosis in the Brugada region starting from previous clinical observations^10,16^. The geometry of our model is inspired by anatomical images^8^. We constructed the geometry considering a portion of the epicardium stretched out on a 2D plane. We represented the pathological region as a semicircle located at one edge of the tissue because it resembles the epicardial impaired substrate (i.e., showing pathological electrograms), as shown in epicardial electrical mapping studies^11^. To quantitatively assess the contribution of electrophysiological and structural alterations to the onset of arrhythmias, we performed multiple simulations for different percentages of fibrotic tissue, $d_w^0$ and size of the BrS region. For the numerical implementation of diffuse fibrosis we adopted the method previously used by Ten Tusscher and Panfilov^35,36^. In particular, we modelled diffuse fibrosis by the presence of inexcitable obstacles of size $1 \times 1$ grid points that were randomly distributed across the tissue. The obstacles had no-flux boundary conditions. We varied the percentage of fibrotic tissue ($F_p$) between 0 and 0.6 with a step of 0.05. We chose the range for the percentage of fibrotic tissue to include the observed values in BrS patients^10^. We varied the value of $d_w^0$ between 0.3 and 0.6 (where 0.6 is the value of healthy myocytes) with a step of 0.05. We did not perform simulations for $d_w^0$ lower than 0.3 because for such values the myocyte is not able to recover the dome, even after several successive excitations. Therefore, we assume that further reduction of $d_w^0$ does not significantly affect the AP morphology and thus the occurrence of arrhythmias. We varied the radius of the BrS region ($R_B$) between 1.5 cm and 4.5 cm with a step of 0.5 cm. The selected size range of the BrS region includes the typical dimensions for the RVOT^8^. Since we randomly distributed the inexcitable obstacles in the BrS region, we carried out 20 simulations for each combination of $F_p$, $d_w^0$ and $R_B$. For each combination of the 3 parameters, we estimated the likelihood of cardiac arrhythmias as the number of observed sustained reentry over the total number of simulations. We ran each simulation for 4 seconds and we recorded sustained reentry if at the end of the simulation there was still a depolarized region in the tissue. Note that due to the random nature of the fibrosis distribution, it is possible that reentry is not consistently observed with the same triplet of parameters. Moreover, when sustained reentry occurred, we evaluated the average frequency of reentry, as the inverse of the average period between two consequent activations of the healthy tissue (recorded in the point (5,8) cm).

## Results

### BrS action potential

**Figure 1:**
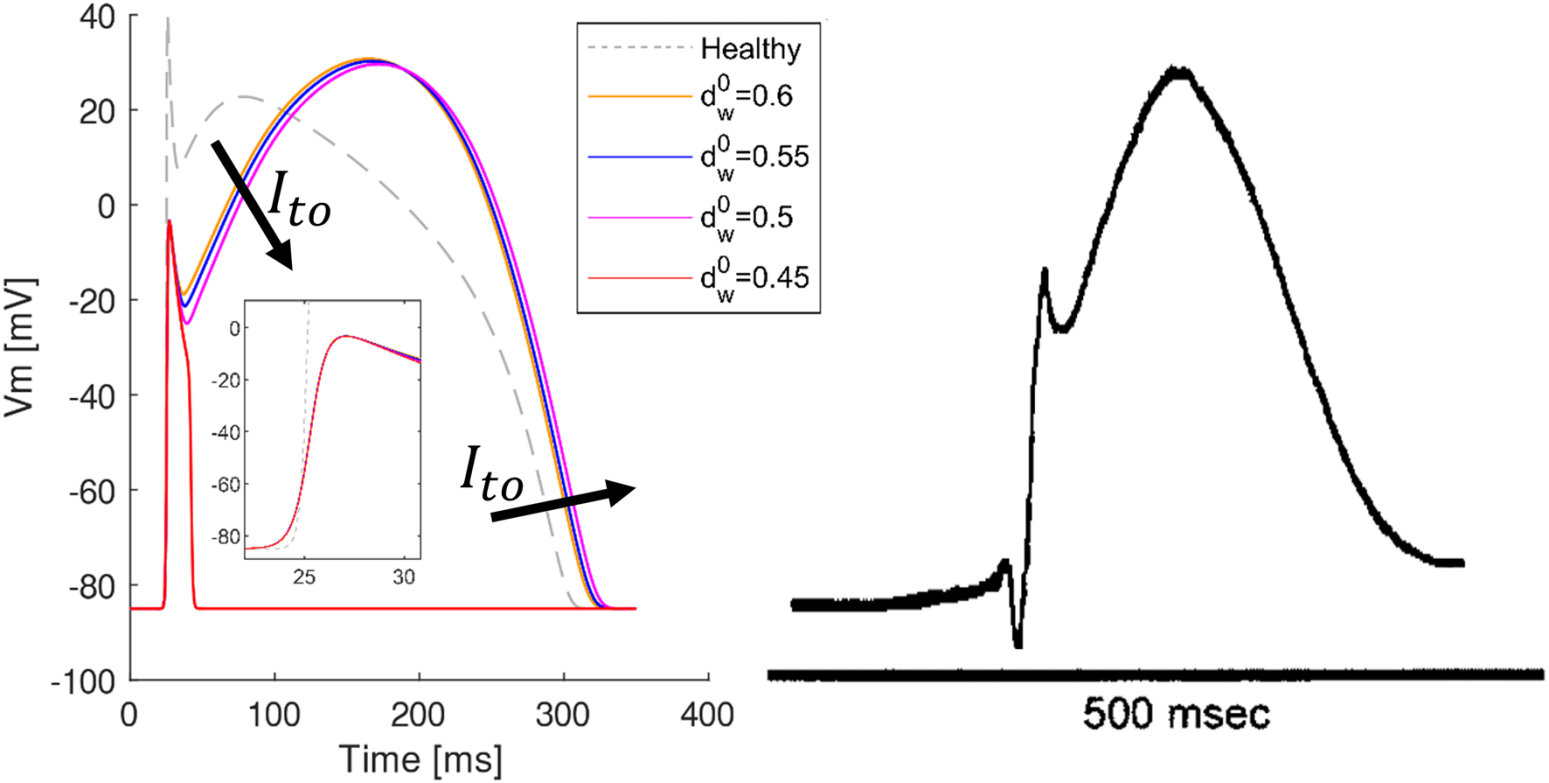
Comparison of simulated and experimental BrS AP. Left: simulated AP for different values of $d_w^0$; arrows indicate the effects of stronger transient outward currents (i.e., lower values of $d_w^0$); the inset zooms on the upstroke phase. Right: in vivo human epicardial monophasic APs^33^.

Figure 1 shows the BrS APs simulated on the 2 cm long homogeneous cable model for different values of $d_w^0$ compared with in vivo human BrS epicardial monophasic action potential recorded in the RVOT^33^. The simulated AP is qualitatively in agreement with in vivo human monophasic action potentials^32,33^ (Fig. 1). For high values of $d_w^0$ (i.e., $d_w^0>0.45$) the transient outward current is not strong enough to completely repolarize the membrane, thus a delayed dome AP is observed. On the other hand, for lower values of $d_w^0$ the transient outward current repolarizes the membrane below the threshold for activation causing a very short AP (i.e., lost dome AP). Both the delay and the loss of the dome have been experimentally observed in canine right ventricular wedge preparations^13,37^. Moreover, for increasing intensity of the transient outward current the APD increases, as previously reported in experimental^32,37^ and computational studies^26,38^. The simulated BrS AP shows an upstroke velocity of 51.4 V/s, which represents about the $25 \%$ of the value obtained in healthy tissue (i.e., 203 V/s). This significant reduction of upstroke velocity matches the results reported by recent studies on induced pluripotent stem cells-derived cardiomyocytes carrying loss-of-function mutation of $Na_v 1.5$^30,31^.

### Mechanism of P2R in 1D model of BrS

As described in “Numerical methods”, we carried out multiple simulations over the 10 cm long heterogeneous cable model to assess the different mechanisms of P2R that may arise when BrS myocytes are coupled with healthy epicardial tissue. As previously reported in several computational studies^25,26,28,29^, P2R can occur in two different modalities: antidromic and orthodromic. In the former the impulse propagating from the BrS region to the healthy region generates a reentrant excitation travelling in direction opposite to the original one. On the contrary, in orthodromic P2R the impulse propagates from the healthy to the BrS region and induces an additional excitation travelling in the same direction.

**Figure 2:**
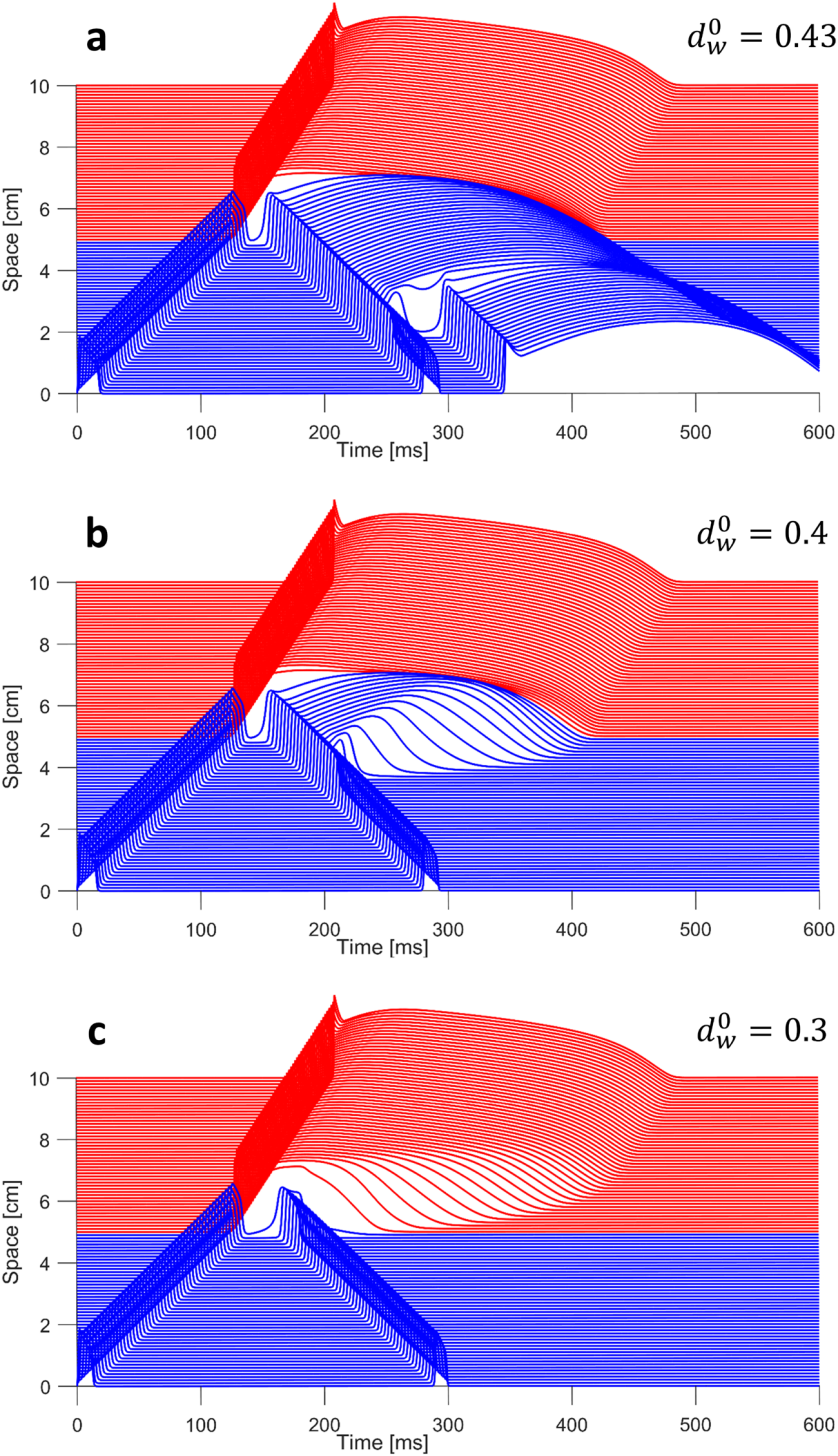
Different antidromic P2R modalities corresponding to different values of $d_w^0$. For each situation, a propagating lost dome impulse in the BrS region (blue) depolarizes the healthy tissue (red). P2R is observed when the electrotonic currents from the healthy region to the BrS region cause a new excitation. (**a**) $d_w^0 = 0.43$; P2R generates a delayed dome AP close to the healthy tissue, whereas farther from the healthy tissue a lost dome AP is followed by a new delayed dome AP. (**b**) $d_w^0 = 0.4$; P2R generates a delayed dome AP close to the healthy tissue, whereas farther from the healthy tissue the AP dome is lost. (**c**) $d_w^0=0.3$; P2R generates a new lost dome AP.

**Figure 3:**
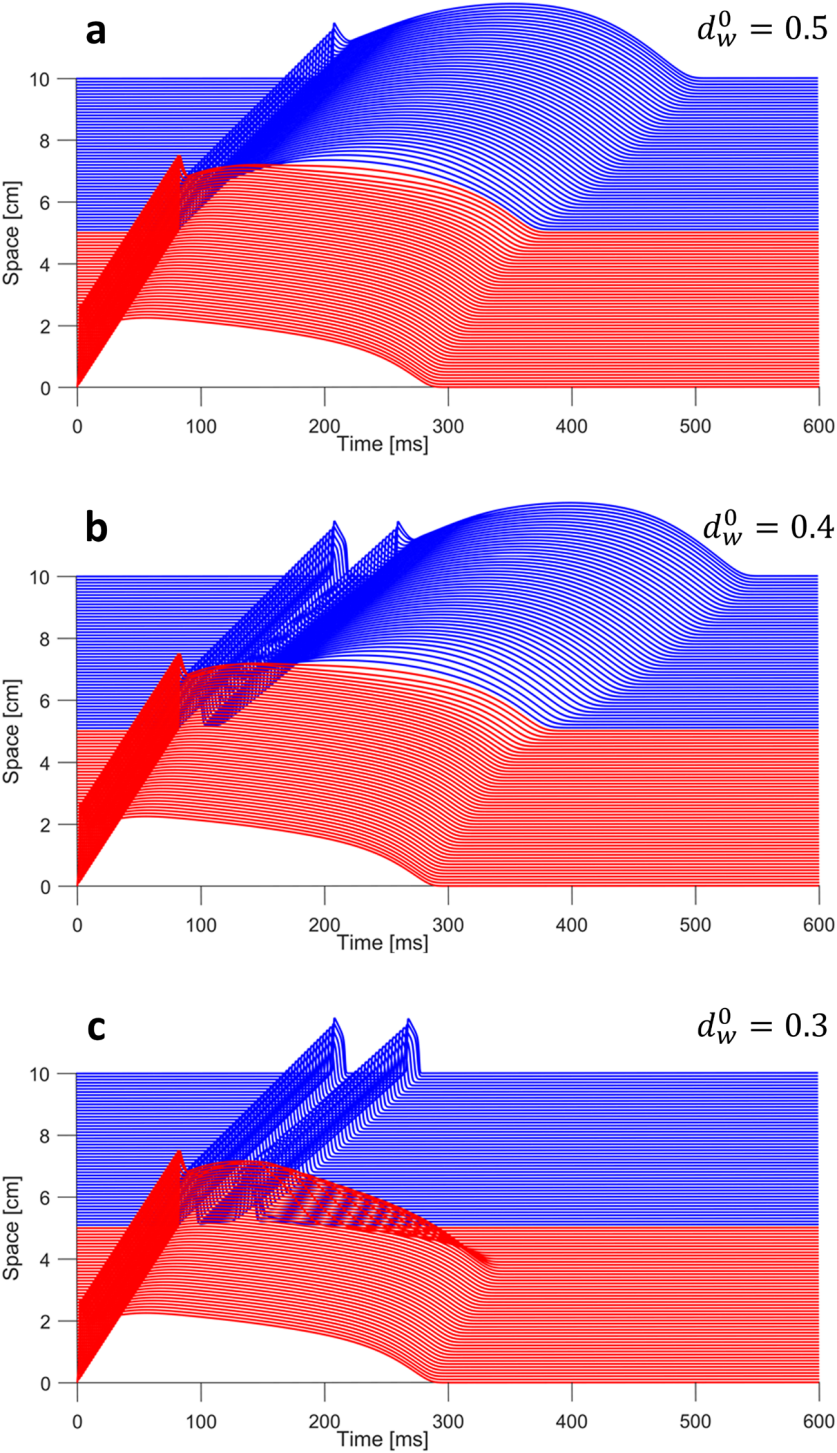
Different orthodromic P2R modalities corresponding to different values of $d_w^0$. For each situation, a propagating healthy AP (red) depolarizes the BrS tissue (blue). P2R is observed when the electrotonic currents from the healthy region to the BrS region cause a new excitation. (**a**) $d_w^0 = 0.5$; a delayed dome action potential propagates in the BrS region, therefore P2R is not possible. (**b**) $d_w^0 = 0.4$; P2R generates a delayed dome AP following the lost dome AP. (**c**) $d_w^0=0.3$; P2R generates a new lost dome AP.

Figure 2 shows some representative examples of antidromic P2R obtained with our 1D model, each one associated with a different value of $d_w^0$. In all the cases, we delivered a single stimulation pulse in the BrS region (blue) and the excitation propagated as a lost dome AP toward the healthy region (red). P2R arises from phase 2 of the AP in regions where the dome is maintained, and propagates into regions where the dome is lost^13,39,40^. Since lost dome APs are particularly short, large voltage gradients may arise between the BrS region and the healthy region. Therefore, if the BrS region recovers while the AP in the healthy region is at its phase 2, electrotonic currents may depolarize again the BrS region initiating a second pulse that propagates in a direction opposite to the original one. Note that if the AP maintains the dome (i.e., $d_w^0>0.45$) P2R is not possible. The morphology of the antidromic AP depends on the value of $d_w^0$. When $d_w^0$ is very close or equal to the limit of 0.45, the antidromic AP shows a delayed dome because the transient outward current has not fully recovered. Therefore, our model formulation can reproduce the two types of APs on the basis of the actual state of the cellular membrane, as other complex ionic models^26,28,29^. Furthermore, our model reflects experimental observations on canine wedge right ventricular preparations where premature beats delivered at relatively low extrastimulus intervals displayed a fully restored dome, whereas premature responses evoked with higher extrastimulus intervals were lost dome^13,41^. For slightly lower values of $d_w^0$, the dome is maintained only for a portion of the cable near to the healthy tissue (Fig. 2a) due to the higher intensity of the transient outward current. Moreover, the portion of the cable where the dome is lost is excited again due to electrotonic coupling with the delayed dome region, thus a new delayed dome AP is generated. As shown in Fig. 2b, further reduction of $d_w^0$ results in a faster loss of the dome of the reentrant AP, without the induction of the additional delayed dome potential. Finally, if the transient outward current is strong enough, the antidromic AP is always lost dome (Fig. 2c).

Figure 3 shows some representative examples of orthodromic P2R, each one associated with a different value of $d_w^0$. In all these cases, we delivered a single stimulation pulse in the healthy region. Orthodromic reentry can be observed when the excitation propagates from the healthy region to the BrS region. Figure 3a remarks that for values of $d_w^0$ higher than 0.45 a delayed dome AP is observed in the BrS, therefore P2R is not possible. When $d_w^0$ is decreased below the threshold of 0.45, a lost dome AP is followed by a second delayed dome AP generated by P2R (Fig. 3b). Finally, for lower values of $d_w^0$ also the reentrant orthodromic AP shows a lost dome morphology (Fig. 3c).

### Mechanism of sustained reentry in 2D model of BrS

**Figure 4:**
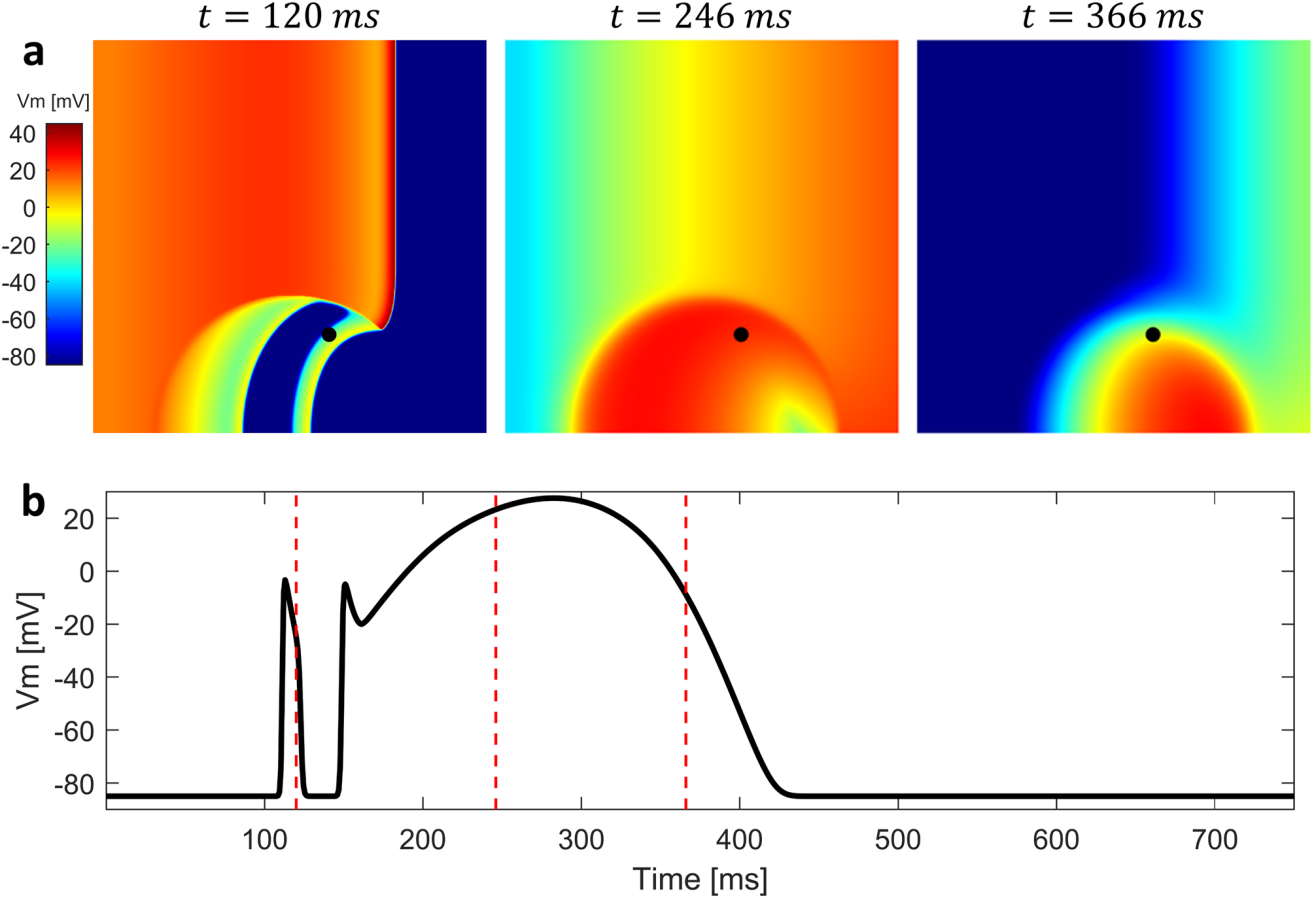
2D simulation with altered electrophysiology in the BrS region without structural abnormalities. (**a**) Sequence of snapshots taken from the $10 \times 10$ cm tissue model. The value of $d_w^0$ was 0.4, whereas the radius of the BrS region was 3.5 cm. Diffuse fibrosis was not present. Sustained reentry was not induced. (**b**) Membrane potential in the BrS region recorded in the point indicated by a black dot in the snapshots. After the first lost dome AP, orthodromic reentry generates a delayed dome AP caused by electrotonic currents from the healthy tissue. Red dashed lines indicate the time instants at which snapshots were taken.

**Figure 5:**
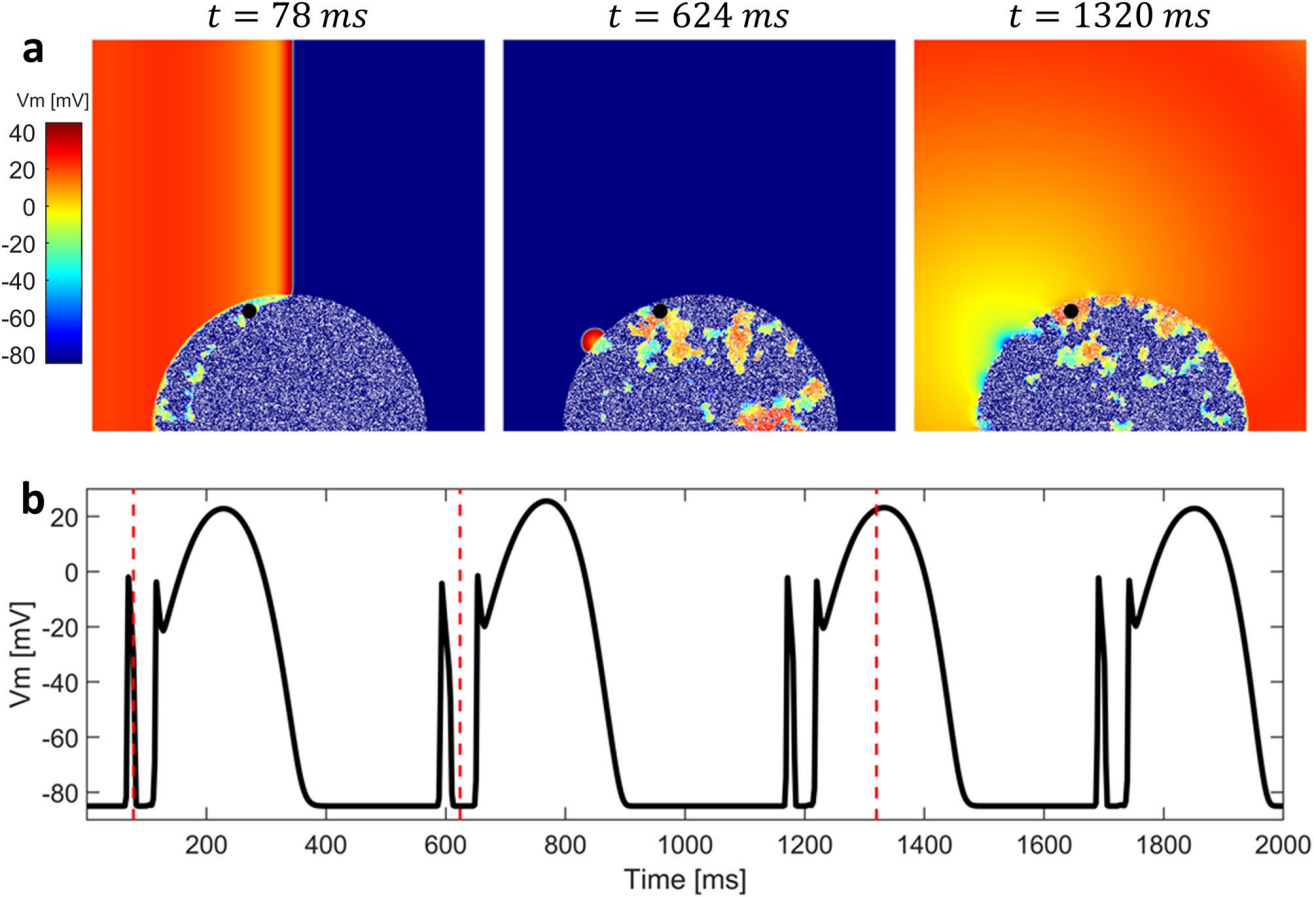
2D simulation with altered electrophysiology in the BrS region with diffuse fibrosis. (**a**) Sequence of snapshots taken from the $10 \times 10$ cm tissue model. The value of $d_w^0$ was 0.4, whereas the radius of the BrS region was 3.5 cm. Percentage of fibrotic tissue was set to $35\%$. Sustained reentry was triggered. (**b**) Membrane potential in the BrS region recorded in the point indicated by a black dot in the snapshots. Both orthodromic and antidromic P2R occurs at the interface between healthy and BrS regions. When diastolic interval is particularly low and/or electrotonic current is particularly high, delayed dome APs are induced. Red dashed lines indicate the time instants at which snapshots were taken.

**Figure 6:**
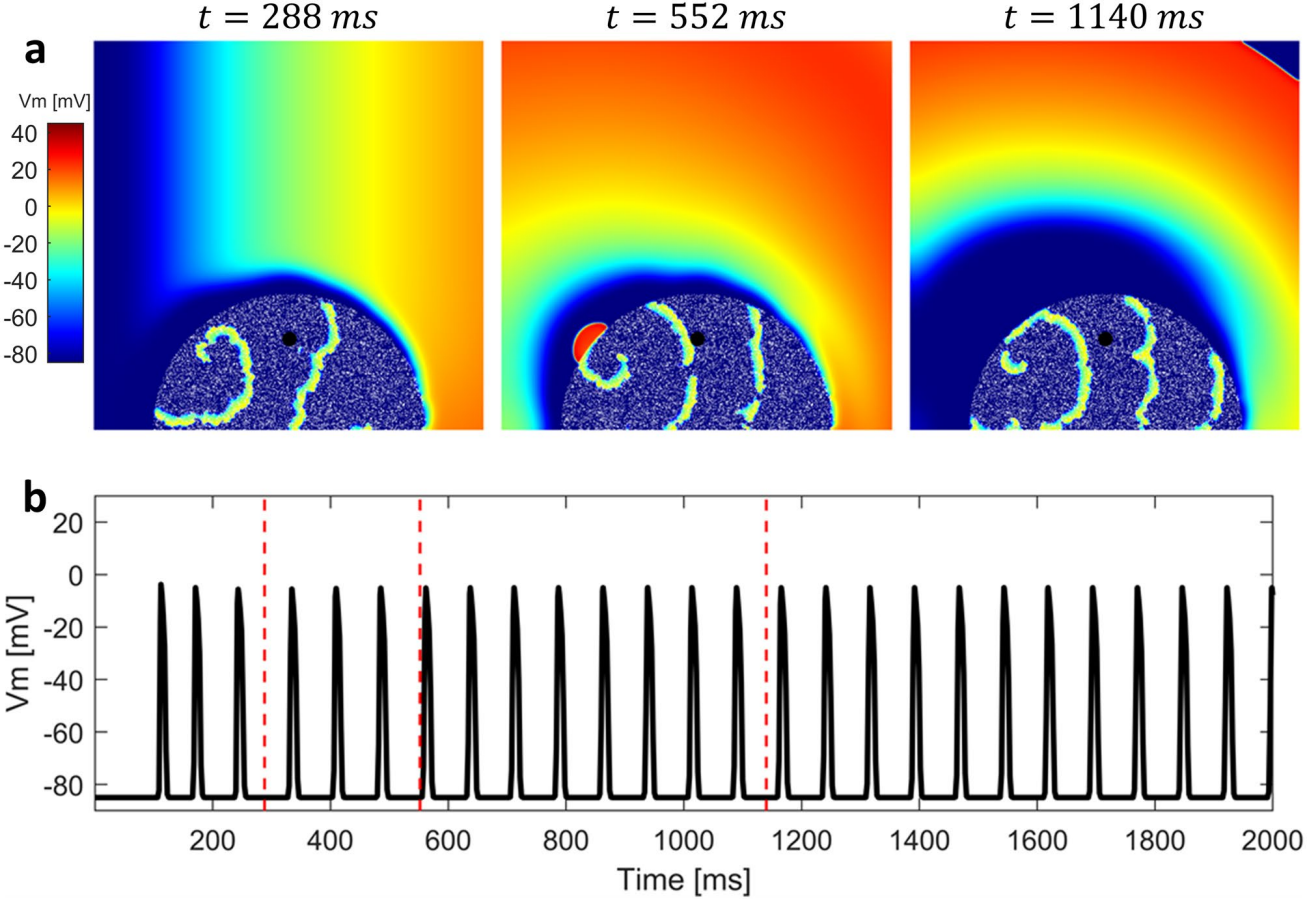
Generation of a lost dome spiral wave in the BrS region reproduced with the 2D model. (**a**) Sequence of snapshots taken from the $10 \times 10$ cm tissue model. The value of $d_w^0$ was 0.3, whereas the radius of the BrS region was 3.5 cm. Percentage of fibrotic tissue was set to $20\%$. A quasi stable spiral wave is observed in the BrS region, and repeatedly stimulates the healthy tissue. (**b**) Membrane potential in the BrS region recorded in the point indicated by a black dot in the snapshots. Both orthodromic and antidromic P2R occur at the interface between healthy and BrS regions. When diastolic interval is particularly low and/or electrotonic current is particularly high, AP is not able to recover the dome. Due to the formation of a quasi stable spiral wave, AP is highly regular. Red dashed lines indicate the time instants at which snapshots were taken.

**Figure 7:**
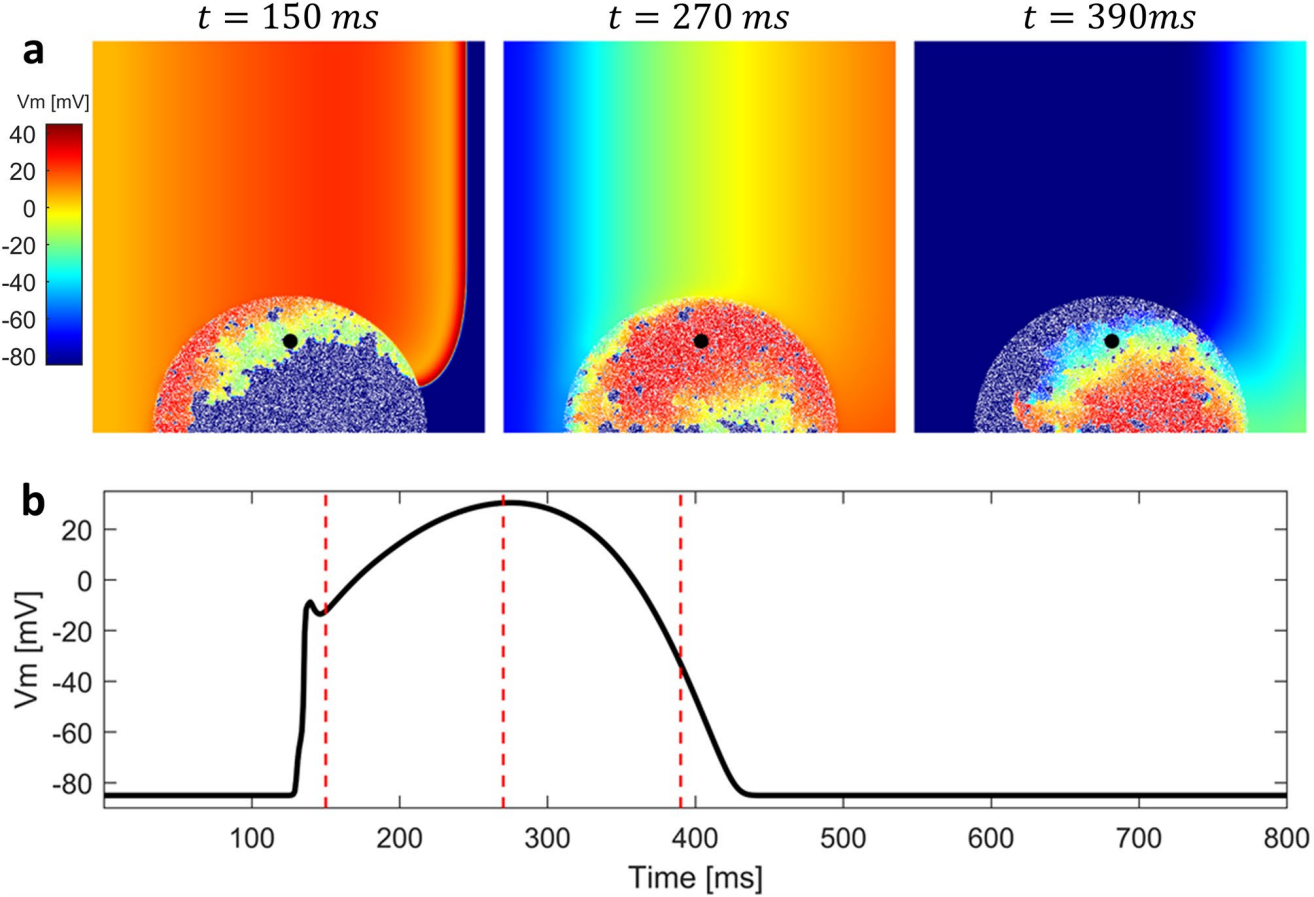
Failure to trigger sustained reentry when AP dome is always maintained. (**a**) Sequence of snapshots taken from the $10 \times 10$ cm tissue model. The value of $d_w^0$ was 0.6, whereas the radius of the BrS region was 3.5 cm. Percentage of fibrotic tissue was set to $35\%$. Sustained reentry was not induced. (**b**) Membrane potential in the BrS region recorded in the point indicated by a black dot in the snapshots. The presence of the dome in the AP prevents orthodromic reentry. Red dashed lines indicate the time instants at which snapshots were taken.

We carried out multiple simulations of the 2D model to assess the different mechanisms of P2R that may arise when both BrS myocytes and fibrosis (in different percentages) are present. For each simulation, we delivered a single stimulation pulse at the left edge of the 2D model, in the healthy tissue. Figure 4a shows three snapshots of the 2D simulation, when diffuse fibrosis was not included in the BrS region (see also supplementary material: Video 1). The value of $d_w^0$ was set to 0.4, whereas the radius of the BrS region was 3.5 cm. Similarly to the one dimensional case, dispersion of repolarization induces orthodromic P2R from the healthy tissue to the BrS region. The newly generated AP shows delayed dome morphology, and has longer duration than healthy AP (Fig. 4b). However, sustained reentry is not induced. Indeed, even if the APD of the delayed dome is higher, the healthy tissue recovers too late in order to be excited again. Sustained reentry is not observed for any combination of $d_w^0$ and $R_B$. Therefore, our results suggest that dispersion of repolarization alone may not be sufficient to induce sustained reentry in our 2D model of human cardiac tissue. In the presence of diffuse fibrosis in the BrS region, sustained reentry can be observed if some conditions are met (Fig. 5 shows a meaningful example, see also supplementary material: Video 2). First, fibrosis reduces the electrotonic coupling between pathological and healthy tissue, thus facilitating loss of dome in BrS region (see supplementary material: Fig. 1 showing the reduction of conduction velocity with the increase of fibrosis percentage). Second, inexcitable discontinuous obstacles, represented by the fibrotic tissue, may lead to breakup of lost dome waves and consequent formation of small reentrant circuits^35,36^ (Fig. 5a). These reentrant circuits can activate again the healthy tissue if they are able to sustain for enough time so that healthy tissue has recovered from its refractory period. Furthermore, healthy myocytes that have been excited by residual activity in the BrS region, may newly depolarize the BrS region in the form of antidromic P2R reentry. Note that under these circumstances, both orthodromic and antidromic P2R are involved in the induction of sustained reentry. Both lost dome and delayed dome APs are observed in the BrS region, similarly to the monodimensional case (Fig. 5b). For lower values of $d_w^0$, the AP is never able to recover the dome (Fig. 6, see also supplementary material: Video 3) facilitating the formation of reentrant circuits. Indeed, the formation of delayed dome regions increases the local wavelength of propagation, thus preventing formation of spiral waves in the BrS region. For high percentages of fibrotic tissue (e.g., $F_p=35\%$ in Fig. 5), spiral waves in the BrS region undergo continuous breakup due to the high density of inexcitable obstacles. Instead, when diffuse fibrosis is less striking, spiral waves in the BrS region are quasi stable and repeatedly stimulate the healthy tissue (e.g., $F_p=20\%$ in Fig. 6a). The formation of quasi stable spiral waves is reflected in the high regularity of the AP (Fig. 6b). According to our simulation, the presence of lost dome APs is essential for the formation of reentrant circuits in the BrS region. As shown in Fig. 7, a delayed dome AP in the fibrotic BrS region is not sufficient to trigger a sustained reentry (see also supplementary material: Video 4). Indeed, when loss of dome does not occur the wavelength is too high for the generation of reentrant circuits in the BrS region.

**Figure 8:**
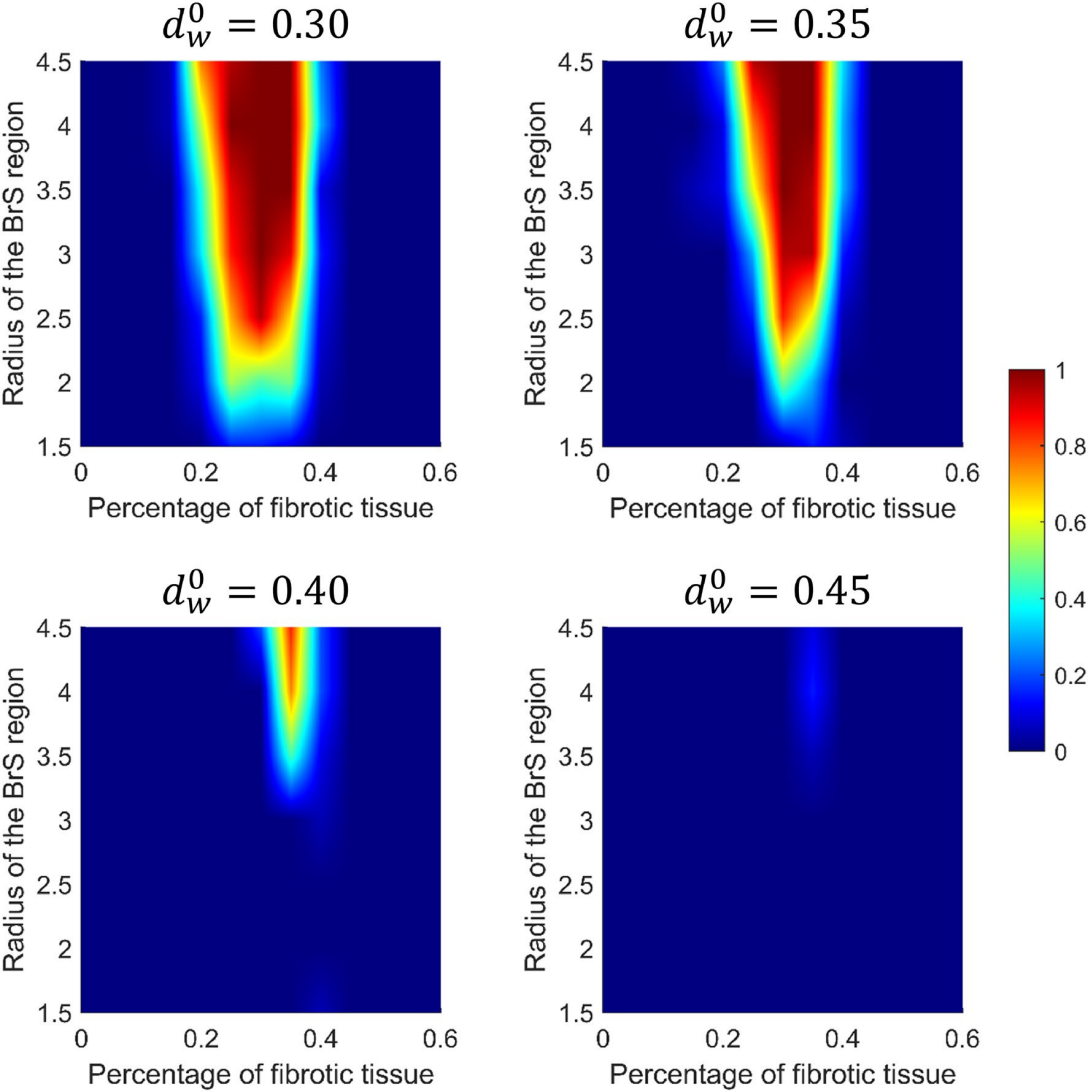
Normalized values of observed sustained re-entries over 20 tissue simulations. The original samples were 13 values of $F_p$ and 7 of $R_B$, the images were linearly interpolated in order to better appreciate the behaviour of the model.

**Figure 9:**
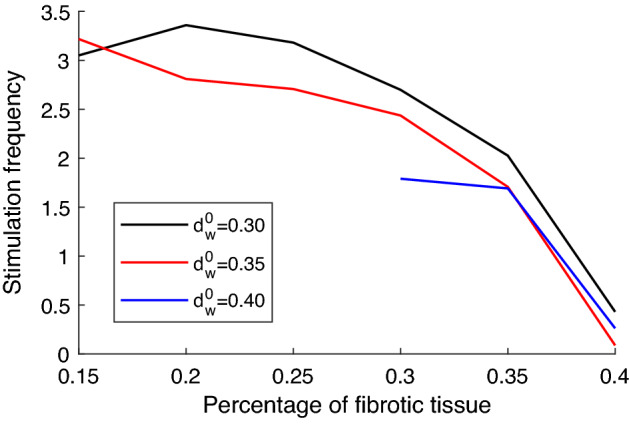
Average frequency of AP in the healthy tissue for different values of $F_p$ and $d_w^0$. Frequency stimulation values were averaged across both the simulation runs and $R_B$. Only the simulations showing sustained reentry were considered.

Figure 8 shows the likelihood of sustained reentry for each combination of fibrosis percentage $F_p$, $d_w^0$ and $R_B$. The results reported in Fig. 8 suggest that the onset of a sustained reentry in BrS may be associated with a specific combination of the size of the BrS region, the fibrosis percentage, and the dynamical properties of the myocyte model. When the percentage of fibrotic tissue was too high ($F_p\ge 45\%$) conduction block between the healthy and Brs region was observed, in agreement with clinical studies^22,23^. In many simulations performed with $F_p$ near to this limit, we observed that reentrant circuits in the BrS region fail to excite the healthy tissue due to current-load mismatch. Therefore, even if residual activity is present in the BrS region, healthy tissue is not involved in arrhythmic events. On the contrary, when the inexcitable obstacles are too few ($F_p<15\%$), wave breakup is not induced, thus formation of reentrant circuits is not possible. Similarly, when the BrS region is too small, even if depolarization waves may break up they are not able to turn on themselves to generate reentrant circuits. Note that there is not an upper limit on $R_B$ for the induction of sustained reentry. Finally, we observed sustained reentry only for values of $d_w^0$ lower or equal to 0.45. Indeed, for $d_w^0>0.45$ BrS AP maintains the dome preventing dispersion of repolarization.

Once we assessed the likelihood of reentry, we evaluated the average frequency of the reentrant activity in the healthy tissue. The frequency for a given simulation was calculated as the inverse of the mean period of an AP observed in the healthy tissue. For each simulation, when sustained reentry was observed, the frequency was averaged. The results are summarized in Fig. 9. The frequency of the arrhythmogenic activity does not depend on the dimension of the BrS region, but only on the percentage of fibrosis present and the value of $d_w^0$. In particular, the frequency of reentry decreases as fibrosis percentage is increased, in agreement with what was observed on previous computational studies on fibrosis-induced reentry^35,36^. Indeed, when $F_p$ is higher the electrotonic coupling is weaker, thus healthy tissue needs to recover for a longer time in order to be excited by the BrS region. On the contrary, when $F_p$ is equal or slightly above $15\%$ the electrotonic coupling between the BrS region and the healthy region is higher, and allows lost dome waves to stimulate the healthy tissue with higher frequency. Whereas the effect of increasing or decreasing the fraction of fibrotic tissue on the frequency of premature ventricular contractions is straightforward, the influence of $d_w^0$ is less intuitive. Our results show that for higher values of $d_w^0$ (lower intensity of transient outward current) the frequency of AP in the healthy tissue is on average lower. This phenomenon is explained by the more frequent occurrence of delayed dome APs in the BrS region. The presence of the dome extends the APD at the interface between the two regions slowing down the recovery of the healthy tissue. As a result, the time between two consequent extra stimulations of the healthy tissue is increased. Note that, when both the percentage of fibrotic tissue and $d_w^0$ are low, the high frequency of stimulation may induce spiral waves with large wavelength in the healthy tissue, similar to spiral waves described in our previous work^24^. Moreover, the average frequency of APs in the healthy tissue achieved with low values of $F_p$ and $d_w^0$ is similar to the dominant frequency of spiral waves in healthy tissue^24^.

## Discussion

To date, there is no clear pathological mechanism commonly acknowledged for BrS. Currently, there are three clinical hypotheses on the pathophysiological mechanisms underlying BrS. Nevertheless, none of these can fully explain both the whole set of clinical observations and the arrhythmic nature related to BrS. In particular, the repolarization hypothesis explains ST elevation in BrS as a consequence of transmural dispersion of repolarization during ST segment^15^. Indeed, the more prominent AP notch, or even the loss of dome, in the RVOT have been shown to produce ST-segment elevation associated with Type I ECG pattern^32,42^. The repolarization hypothesis is also consistent with the arrhythmic behaviour of BrS. Arrhythmic events may be triggered by P2R originating from zones where the dome is maintained to regions where the dome is lost, as shown in canine RV wedge preparations^13^. However, it is worth mentioning that canine experimental models have some limitations. First, the electrophysiological properties of animal cardiac tissue are different from human cardiac tissue^43–45^. Moreover, in experimental animal models BrS is pharmacologically induced in coronary perfused ventricular wedges, therefore reproducing the pathological substrate of the RVOT is challenging^46^. Szèl and Antzelevitch^14^ used a canine RV wedge preparation to explain electrogram fractionation and late potentials. This preparation did not include structural abnormalities and conduction disturbances. The study demonstrated the ability to generate secondary deflections on the epicardial electrograms based on differences in the AP morphology at different epicardial locations during the AP repolarization phase. However, in this case, deflections are generated by AP upstrokes of phase 2 reentrant beats and not by structural abnormalities. Therefore, fractionation includes at most two late deflections occurring with a minimum delay of about 100 ms. Nevertheless, as shown by Zhang and colleagues^47^, electrogram fractionation in BrS patients has more than 2 deflections with a coupling interval lower than 50 ms from the main deflection. Furthermore, in BrS patients deflections can be seen also before the main deflection, and the fractionation is amplified with increased heart rate. Finally, according to the repolarization hypothesis, conduction delay, typically observed in BrS patients^16,17^, may be explained by the reduced upstroke velocity caused by reduction of sodium currents^6^. The depolarization hypothesis states that conduction delay and disruption of the AP propagation in the RVOT are responsible for the ST segment elevation^16,17^. As a matter of fact, cardiac structural abnormalities are commonly observed in BrS patients and may even cause excitation failure via current-load mismatch, which is the third hypothesis concerning ST elevation^18,19^. The presence of structural abnormalities easily explains electrogram fractionation and conduction delay^10^. However, it does not provide a triggering mechanism for the induction of sustained reentry.

In this study, we developed a computational 2D model of BrS, including both the RVOT affected tissue and healthy epicardial tissue. We employed our model to assess the contribution of the different arrhythmogenic factors underlying BrS in the development of arrhythmic events. Our computational study suggests that structural abnormalities are essential to induce sustained reentry. Indeed, when diffuse fibrosis is not considered in our model, the phase-2 reentrant AP is not long enough to stimulate again the healthy tissue, similarly to the “concealed” P2R observed by Szèl and Antzelevitch^14^. Furthermore, when repolarization abnormalities (i.e., loss of dome) are not included in the BrS region of our model, arrhythmic events are not observed. In truth, without repolarization abnormalities the human cardiac wavelength is too large to allow generation of small reentrant circuits in the BrS region^36^. Instead, according to our simulations, sustained reentry can occur only when the BrS region is characterized by both diffuse fibrosis and loss of AP dome. Therefore, our computational study suggests that the arrhythmic substrate of BrS is determined by both depolarization and repolarization abnormalities. Finally, by increasing the percentage of fibrotic tissue we were also able to reproduce conduction block, as recorded in several clinical studies^18,47^. The reentry mechanism we proposed for BrS is similar to the reentry near the percolation threshold reported by Alonso and Bär^48^, although our model employs two different electrophysiological formulations for the fibrotic and non fibrotic regions. Reentry near the percolation threshold has been already investigated as a mechanism underlying cardiac arrhythmias, such as atrial fibrillation^49^. The site percolation threshold for a 2D square lattice is 0.41^48^. Our simulations are coherent with percolation theory, showing the highest probability of reentry slightly below the percolation threshold. Indeed, formation of microreentries in our model is observed with highest probability near to this limit. Note that total probability of reentry is higher in our case with respect to the results obtained by Alonso and Bär^48^, since lost dome AP has reduced wavelength. Moreover, increasing $d_w^0$ results in an average longer wavelength and reduces the probability of reentry, as noted by Alonso and Bär, especially for lower values of fibrosis. In addition, increasing the size of the BrS region increases the probability of reentry homogeneously with respect to percentage of fibrotic tissue, again in agreement with the results by Alonso and Bär^48^.

Other computational studies dealt with the arrhythmic nature of BrS, mainly focusing on P2R as the cause of arrhythmogenesis^25–27^. Bueno-Orovio et al. used a phenomenological model to investigate the tissue-level reentrant mechanism of BrS as a repolarization disorder^25^. Their computational study on an epicardial tissue layer without structural abnormalities shows that sustained reentry can arise when three regions corresponding to delayed-dome (i.e., where AP morphology reproduces recordings from an experimental canine model), lost-dome, and normal epicardium are considered. However, the spatial distribution of the three regions does not reflect the coupling between BrS and healthy tissue observed in the RVOT. In addition, the APD in the healthy epicardium is shorter than normal. Note also that, differently from our model and from experimental studies^13,41^, the Bueno–Orovio phenomenological model does not lose the AP dome depending on the membrane state: it either always loses the dome or always shows the delayed dome AP. Thus, delayed dome and lost dome AP are associated with two different sets of parameters and need to be collocated in the tissue model. Cantalapiedra et al.^26^ employed the Luo–Rudy model^50^ of epicardial ventricular action potential to study the role of transient outward current heterogeneities and sodium inactivation kinetics in the reexcitation mechanism. It must be noted that the Luo–Rudy model is based mostly on guinea pig ventricular cell data, making it difficult to generalize the obtained results to human cardiac tissue. Heterogeneities in the transient outward current were introduced by defining two regions with different values of the transient outward conductance ($g_{to}$), whereas the sodium kinetics was modified independently in the whole tissue. Notably, the myocyte model used by Cantalapiedra et al. is able to reproduce both delay and loss of the AP dome depending on membrane state. The results showed the formation of sustained reentry in a two-dimensional epicardial tissue with modified sodium kinetics and heterogeneous transient outward current. However, the area in which the APs keep the dome (i.e., the region with lower $g_{to}$) must be such as to break the symmetry, in order to generate reentry (e.g., with a triangular shape). Also, the region with lower $g_{to}$ is surrounded by a bigger region of tissue with higher $g_{to}$, where the dome is often lost. Furthermore, coupling with healthy tissue was not considered, thus facilitating reexcitation in the form of P2R. The results from previous computational studies have provided valuable insights in the pathophysiological mechanism of BrS and P2R, however these studies neglected the experimental observations on the nature of the arrhythmic substrate. In fact, many experimental studies identified the RVOT as the principal target for both the BrS action potential and structural abnormalities^16,47^. Nevertheless, none of the aforementioned computational studies modeled a region resembling the RVOT.

## Conclusion

In this work, we carried out a computational study assessing the contribution of the different arrhythmogenic factors underlying BrS in the development of arrhythmic events. Supported by previous experimental studies^10,19,22,41^, we propose an arrhythmic mechanism that unifies the repolarization and depolarization hypothesis of the pathophysiology of BrS. In particular, our model considers a finite region with both structural and electrophysiological alterations resembling the RVOT, as experimentally observed in BrS patients^8,10,16^. The BrS region was enclosed in a bigger region representing healthy tissue. We observed that both structural and electrophysiological abnormalities are necessary to generate P2R, giving a common ground for repolarization and depolarization hypotheses. Indeed, our results suggest that dispersion of repolarization is not sufficient to induce sustained reentry in human tissues.

Nevertheless, our work has some limitations. Clearly, while 2D results may give important insights on the arrhythmic mechanism of BrS, a simulation on a 3D ventricular geometry, including endo-, mid-, and epicardial cells would be more realistic. Our future works aim to overcome this limitation and extend our observations on a topologically accurate model, including fiber anisotropy. Simulations on 3D topologically accurate models will also be useful for the comparison with simulated and experimental electrograms obtained from the RVOT of BrS patients. Furthermore, in this study we only considered diffuse fibrosis as the source of structural abnormalities. While the modeling of fibrosis with $1 \times 1$ inexcitable cells is well established in the literature^35,36,51^, it may overlook physiological mechanisms which may be relevant to the insurgence of cardiac arrhythmias. In particular, the size and shape of inexcitable obstacles may be important in determining the arrhythmic substrate associated with BrS^52^. Therefore, in future works we wish to consider different patterns of fibrous deposits^53^ and study how the size and shape of inexcitable obstacles affect the reentrant mechanism. Moreover, we wish to explore different options for fibrosis representation, such as the edge splitting method, and the additional effect of myocyte-fibroblast coupling^54^.

As a last consideration, we did not consider the effect of pacing frequency on the proposed reexcitation mechanism. However, since APs tends to recover the dome at higher stimulation rates, we expect that the likelihood of sustained reentry decreases as the stimulation period decreases. This trend was already observed in a previous 1D computational study^26^ and is in agreement with the clinically observed fact that sudden cardiac death in patients with BrS has higher incidence during sleep, when cardiac rhythm is lower^2,55^. Thus, we deem necessary to include the pacing frequency as a model parameter in future works.

## Supplementary Information

Supplementary Information 1.

Supplementary Information 2.

Supplementary Information 3.

Supplementary Information 4.

Supplementary Information 5.

Supplementary Information 6.

## Data Availability

The data generated by the simulations of this study are available upon request from the corresponding author.
