# Supplementary figures and images for: Diffuse fibrosis and repolarization disorders explain ventricular arrhythmias in Brugada syndrome: a computational study
Source: Sci Rep. 2022 May 20;12:8530. doi: 10.1038/s41598-022-12239-9 (PMC9123016; doi:10.1038/s41598-022-12239-9)

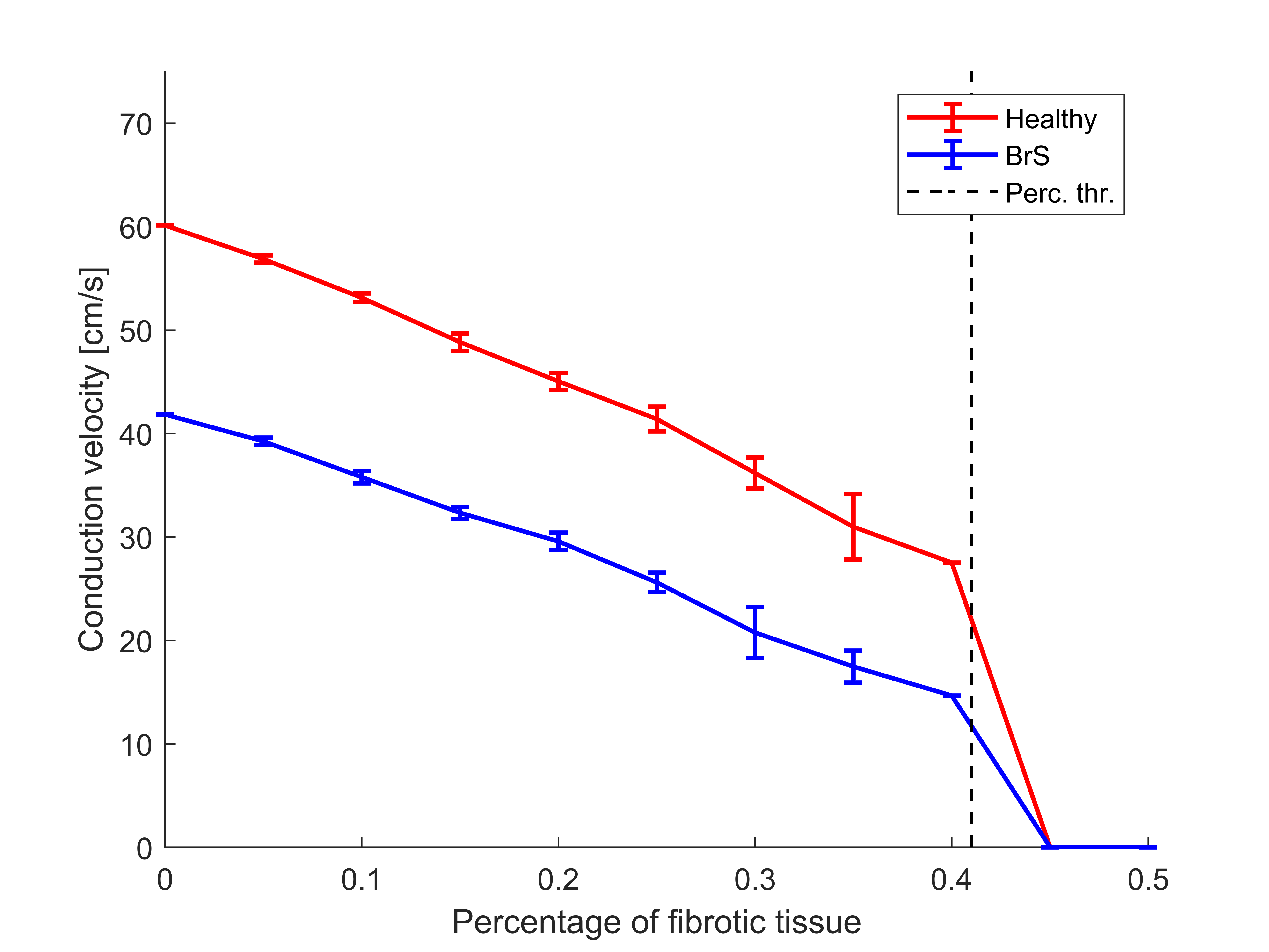

Supplement: Supplementary file 5 — Supplementary Information 5. [file 41598_2022_12239_MOESM5_ESM.png]
