# Supplementary material for: Diffuse fibrosis and repolarization disorders explain ventricular arrhythmias in Brugada syndrome: a computational study
Source: Sci Rep. 2022 May 20;12:8530. doi: 10.1038/s41598-022-12239-9 (PMC9123016; doi:10.1038/s41598-022-12239-9)
Supplement: Supplementary file 6 — Supplementary Information 6. [file 41598_2022_12239_MOESM6_ESM.pdf]

Diffuse fibrosis and repolarization disorders explain ventricular arrhythmias in Brugada syndrome: a computational study

Description of the videos and figures uploaded as supplementary material.

VIDEO S1:
2D simulation with altered electrophysiology in the BrS region without structural abnormalities.
Top: The video shows the 10x10 cm tissue model with excitation delivered from the left edge. The value of dw0 was 0.4, whereas the radius of the BrS region was 3.5 cm. Diffuse fibrosis was not present. It can be seen that sustained reentry was not induced.
Bottom: Membrane potential in the BrS region recorded in the point indicated by a black dot in the model. After the first lost dome AP, orthodromic reentry generates a delayed dome AP caused by electrotonic currents from the healthy tissue.

VIDEO S2:
2D simulation with altered electrophysiology in the BrS region with diffuse fibrosis.
Top: The video shows the 10x10 cm tissue model with excitation delivered from the left edge. The value of dw0 was 0.4, whereas the radius of the BrS region was 3.5 cm. Percentage of fibrotic tissue was set to 35%. Sustained reentry was triggered.
Bottom: Membrane potential in the BrS region recorded in the point indicated by a black dot in the snapshots. Both orthodromic and antidromic P2R occurs at the interface between healthy and BrS regions. When diastolic interval is particularly low and/or electrotonic current is particularly high, delayed dome APs are induced.

VIDEO S3:
Generation of a lost dome spiral wave in the BrS region reproduced with the 2D model.
Top: The video shows the 10x10 cm tissue model with excitation delivered from the left edge.The value of dw0 was 0.3, whereas the radius of the BrS region was 3.5 cm. Percentage of fibrotic tissue was set to 20%. A quasi stable spiral wave is observed in the BrS region, and repeatedly stimulates the healthy tissue.
Bottom: Membrane potential in the BrS region recorded in the point indicated by a black dot in the video. Both orthodromic and antidromic P2R occur at the interface between healthy and BrS regions. When diastolic interval is particularly low and/or electrotonic current is particularly high, AP is not able to recover the dome. Due to the formation of a quasi stable spiral wave, AP is highly regular.

VIDEO S4:
Failure to trigger sustained reentry when AP dome is always maintained.
Top: The video shows the 10x10 cm tissue model with excitation delivered from the left edge. The value of dw0 was 0.6, whereas the radius of the BrS region was 3.5 cm. Percentage of fibrotic tissue was set to 35%. Sustained reentry was not induced.
Bottom: Membrane potential in the BrS region recorded in the point indicated by a black dot in the snapshots. The presence of the dome in the AP prevents orthodromic reentry.

FIGURE S1: Averaged conduction velocity as a function of the percentage of fibrosis for healthy and Brugada tissue. Error bars indicate standard deviation over 10 trials. The dashed line represents the percolation threshold.
